# Supplementary material for: Magnetic Field-Assisted Orientation and Positioning of Magnetite for Flexible and Electrically Conductive Sensors
Source: Micromachines (Basel). 2025 Jan 8;16(1):68. doi: 10.3390/mi16010068 (PMC11768084; doi:10.3390/mi16010068)
Supplement: Supplementary file 1 [file micromachines-16-00068-s001.zip › micromachines-3401383-supplementary.pdf]

micromachines

Supplementary Materials

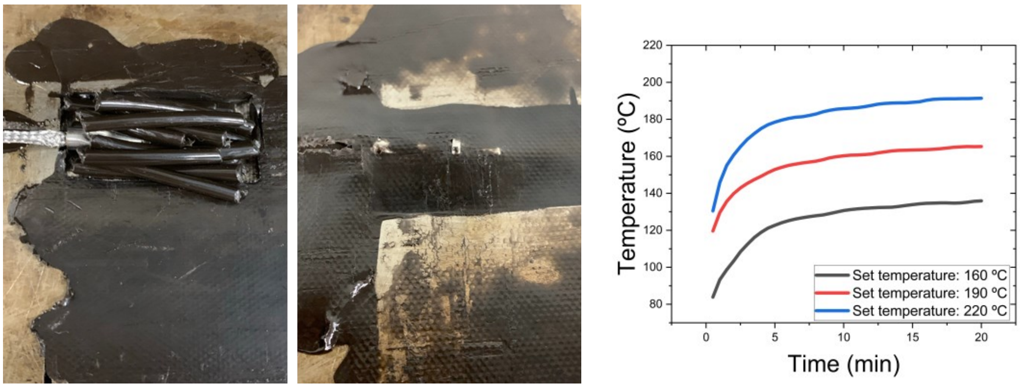

(a) (b) (c)

Figure S1. Temperature monitoring on the compression mould cavity. (**a**) sensor integration on the mould cavity. (**b**) sensor inside the molten polymer. (**c**) temperature profile for each set temperature for 20 min.

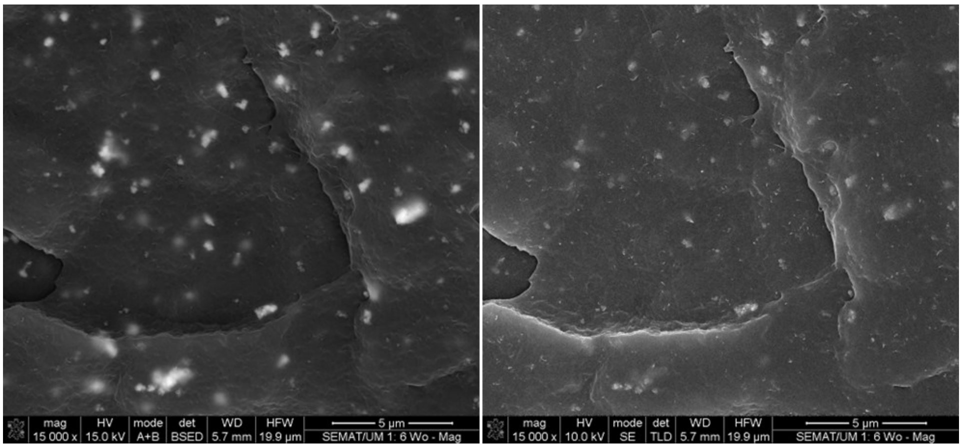

(**a**) (**b**)

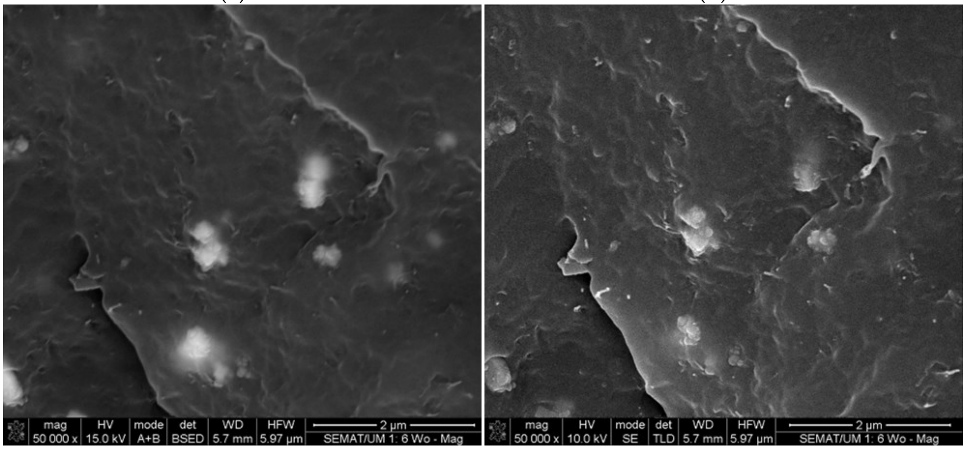

(**c**) (**d**)

Figure S2. 1% MWCNT 6% magnetite without magnetic field in (**a**) BSE and (**b**) SE at and (**c**) BSE and (**d**) SE.
